# 2438 Community-based research networks: Providing infrastructure for clinical and translational research in the State of Michigan

**DOI:** 10.1017/cts.2018.241

**Published:** 2018-11-21

**Authors:** Meghan Spiroff, Patricia Piechowski, Karen D. Calhoun, Susan Goold, Ayse Buyuktur, Michael Klinkman, Elias M. Samuels, Zachary Rowe, Zachary Rowe

**Affiliations:** University of Michigan School of Medicine

## Abstract

OBJECTIVES/SPECIFIC AIMS: As the sole Clinical and Translational Science Award (CTSA) site in Michigan, the Michigan Institute for Clinical & Health Research (MICHR) at the University of Michigan (UM) is working to develop community networks that drive clinical and translational research on community-identified health priorities. METHODS/STUDY POPULATION: These CBRNs will be modeled from successful work that has been accomplished in Jackson, MI where stakeholders from the local healthcare community, County Health Department, Health Improvement Organization, and grassroots community members created a Community of Solution to address the unmet behavioral health and social needs of community members. The CBRN’s will focus on identifying community health priorities by receiving input from community members in underserved communities using deliberative software called Choosing All Together (CHAT). RESULTS/ANTICIPATED RESULTS: In the fall of 2017, 3 focus groups were held in Northern Michigan to identify community health priorities. The top 5 community health priorities include; (1) mental wellness, (2) long-term illness, (3) alcohol and drugs, (4) air, water, and land, and (5) affording care. Additional focus groups are scheduled for the winter in 2 additional geographic areas. DISCUSSION/SIGNIFICANCE OF IMPACT: Future work for the creation of CBRNs includes building leadership groups comprised of clinicians, community leaders, public health leaders, health system leaders and researchers to inform the leadership groups of community-identified health priorities. In addition, the team is working to identify a platform to connect academic investigators across UM and community partners on shared research priorities in real time. In order to measure and map relationships within the networks, we are planning to utilize Social Network Analysis as an evaluation tool.

